# Investigation of The Apoptotic and Antiproliferative Effects of Boron on CCL-233 Human Colon Cancer Cells 

**DOI:** 10.22074/cellj.2021.7259

**Published:** 2021-08-29

**Authors:** Şahabettin Can Özyarım, Funda Karabağ Çoban

**Affiliations:** 1 Institute of Science, Department of Molecular Biology and Genetics, Usak University, Usak, Turkey; 2 Department of Molecular Biology and Genetic, Faculty of Science and Art, Usak University, Usak, Turkey

**Keywords:** Boric Acid, Colon Cancer, Poly (ADP-) Ribose Polymerase, Proliferation, Vascular Endothelial Growth Factor

## Abstract

**Objective:**

Colorectal cancer is one of the most prevalent consequences of cancer-bound decease worldwide and it remains one of the leading outcomes of cancer-bound decease. Boron is an important mineral that acts significant function in various biological courses. Some important chemical properties of boric acid support its utility in the treatment of cancer. The aim of this study is to evaluate the antiproliferative effects of boric acid in colon cancer.

**Materials and Methods:**

This experimental study effect of different concentrations of boric acid on the CCl-233 human colon adenocarcinoma cell lines was investigated, by analyzing proliferation assay (proliferation was applied to the cells for 24, 48 and 72 hours). Proliferation assay was performed using CCK8 Assay Kit. Vascular endothelial growth factor (VEGF) and poly (ADP-) ribose polymerase (PARP) analyses were performed using Sun-Red Human (VEGF) ELISA Kit and Sun-Red Human (PARP) ELISA Kit, respectively.

**Results:**

As a result of the studies, analysis of the cell viability showed that 50 mM boric acid decreased cell proliferation after 24, 48 and 72 hours. The maximal decrease in cell proliferation was found to occur at 48 hours. Therefore, PARP and VGEF analyses were performed at 48 hours. PARP values were significantly higher in cisplatin (P<0.05). In contrast, PARP levels were significantly lower (P<0.05) at two concentrations of boron (50-100 mM). In VEGF, analysis showed that boron levels were significantly different from cisplatin, but there was no significant difference between control groups.

**Conclusion:**

It is proposed that the molecular mechanisms leading to this type of cancer as well as the effect of boric acid on colon cancer should be clarified in more detailed ways for the early diagnosis and treatment of colon cancer.

## Introduction

Cancer is a fatal disease developing by rapid and uncontrolled cell division. When such abnormal development occurs in the colon or rectum, it is considered colorectal cancer (CRC), which is characterized as colon cancer. Colon (large intestine) is approximately 5 feet long in the digestive tract and absorbs water and salts from food. Rectum is a muscular tube that reaches 6 inches of the digestive system (1).

CRC is a malignancy that occurs in one of the colon tissues representing the longest branch of the intestine and rectum tissues, as the majority part of intestine in front of the anus. CRC is the most important reason of cancer decease worldwide. Almost all CRC starts as a small development called polyp; such polyps are typically benign and they can grow like cancer after a few years, but this can last more than 10 years. Colorectal cancer is the second most widespread cancer in male and the third most widespread cancer in the female population (2).

Occurrence of CRC is widely different across the globe and is most common in developed countries, particularly in Australia, New Zealand, Europe and North America. The minimal incidences of this disease are in Africa and Asia. These geographical inconsistencies can be cited as variances in diet and environmental factors. However, the incidence of CRC in new developed countries is increasing. The increase in the incidence of cancers can be attributed to westernization of the diet, increased obesity, and inactivity (3).

Several factors have been associated with CRC risk, including age, polyp presence, inflammatory bowel disease, standard of living, and history of genetic impairment. Environmental factors such as obesity, physical inactivity, malnutrition, smoking and excessive alcohol use account for approximately 80% of all CRC conditions (4). Emerging CRC may not show any symptoms, and CRC indications often rely on the ability and position to metastasize (5, 6).

Most of the drugs currently used for cancer treatment are cytotoxic drugs that interfere with the functioning of cell DNA. Recognition of cytotoxic compounds within ten years caused improvement of anticancer therapy. Although cancer treatment has been advanced, it has been limited to identify unparalleled biochemical directions of malignities that can be used to select destination of tumor cells (7).

Boron is a mineral matter found in nature and it is prevalently used in health system. It is quickly absorbed after enforcing to the body and pervades quickly owing to passive diffusion (8). Although there are not many studies reported to determine the effects of boron on humans, existing studies have not shown adverse effects in drinking water and normal exposure to food (9, 10). According to World Health Organization (WHO) standards, boron and the corresponding compounds are non-toxic, although the limit value in drinking and potable water is 0.3 mg/L, especially boric acid and sodium borates which have antiseptic properties (11). Boron is found in water, soil and plants. Due to the bone health, it advised to intake 3 mg/ day boron (12). WHO reported a reliable intake range of 1-13 mg/day for adults. Boron insufficiency is not frequent in humans. evidences showed that boron deficiency in humans is <1 mg/day intake per day for 63 days. Available information on deadly levels of boron is scant. Boron is an important element for life, receive dissimilar resources from body. Toxicologial impacts of boron and the respective compounds over body, have not been studied sufficiently particularly at tissue level. Toxicity of oral route boron intake is low. Boron is a mineral element that is rapidly absorbed and excreted from the kidneys. It is reported that boron levels in blood, tissue and urine are increased due to systemic toxic effects of boric acid and borax in experimental animals and humans. The highest level of boron accumulation is in brain, liver, kidney and blood (13). Boron has impacts on calcium and potassium metebolisms (14), cyt B5 reductase (15, 16), insulin, estrogen, testosterone, triiodothyronine, thyroxine (17) and reactive oxygen species (ROS) metabolism (18, 19). At the same time, biochemical mechanism of boron is not exactly known yet. Now, two conjectures are improved for biochemical functions of boron in animals and humans. First, boron might play role in cell membrane functions that impact in reply to hormone action, trans membrane signaling and trans membrane move of regulative ions (20). Second, boron might task as a metabolic trimmer in various enzymatic-systems (21). 

Boric acid and borax are the most important of compounds of boron (22). Boric acid (H_3_ BO_3_ ) is the most common seen form of boron in beasts and humans. It has no color and odor, easily soluble in water due to the transparent crystal structure. Various biological functions of boron compounds are known. Boron supplementation leads to increased psychomotor speed and dexterity as well as short-term cognitive attention and memory processes (23- 25). Boron restraints oxidative injury by increasing body depot of gulutathione and different structures or stimulating another ROS neutralizing agents (26). Some important chemical properties of boric acid support its utility in treatment of cancer. Experimental and epidemiological studies have shown that boric acid had a positive effect on human prostate cancer cells. Depending on the concentration of boric acid, NAD in cancer cells and Ca^++^ release. It was reported that proliferation of cancer cells were decreased by borates. These anticarcinogenic effects of boron may be related to its effect on Nicotinamide adenine dinucleotide and calcium channel (27). Boron, existenting especially in the structure of bones and teeth, plays crucial role in the absorbtion of calcium, phosphorus and magnesium. Therefore, it is a vital element for bone health. 

The aim of this study was to determine impacts of different boron concentrations in CCL-233 cells. For this purpose, were evaluated the effects of boron on proliferation, apoptosis and angiogenesis.

## Materials and Methods

### Materials

Boric acid is a source of boron and Cisplatin purchased from Sigma-Aldrich (Interlab, Turkey). All the other chemicals and reagents were of the analytical reagent grade purchased from SunRed Biotechnology. 

*In vitro* cell culture, as part of this study, was carried out in Usak University (Usak, Turkey) Scientific Analysis and Technological Application and Research Center, Cell Culture Research Laboratory. Human colon cancer cells used in the study (CCL-233-SW-1116), was obtained from Manisa Celal Bayar University (Manisa, Turkey) cell culture laboratory.

### Cell culture study

Our studies were carried out* in vitro* and this is an experimental study. It was performed in Uşak University, Scientific Analysis and Technological Application and Research Center by using cell culture research laboratory facilities.

The medium for CCL-233-SW-1116 cells was prepared as 90% Roswell Park Memorial Institute-1640 (RPMI-1640, with L-Gln, Thermofisher Scientific ABD, United States), 10% fetal bovine serum (FBS, Thermofisher Scientific ABD, United States), 1% penicilin-streptomycin (Thermofisher Scientific ABD, United States). The cells were cultured and passaged in 25 cm² and 75 cm² flask containing the indicated medium, in 37˚C incubator containing 5% CO_2_ . Laminar flow cabinet was used for cell passages, inverted microscope was used for cell culture investigations and -80˚C deep freezer was used for storage of the cell stocks.

Our study was accepted by The Butler University Institute of Science decision number 2019/197.

### Proliferation of cells

The cells were sub-culture when they reached 80- 90% density in 4 ml RPMI-1640 (included L-glutamine, Thermofisher Scientific ABD, United States) medium in 25 cm² flask and 12 ml 10% FBS and 1% penicillin-streptomycin in 75 cm² flas

### Passaging cells

By reaching to 80-90% density, the cells were ready for new passage. To passage cells media was first removed. Next, 8 ml and 24 ml phosphate buffer saline (PBS, Thermo Fisher Scientific, USA) was added to the respectively 25 cm² and 75 cm² flasks. Dead cells was removed Upon discarding PBS. To each 25 cm² flask, 2-2.5 ml trypsin (Capricorn, Germany, CP18-2312) (0.25%) was added and 5-7 ml trypsin was added to each 75 cm² flask followed by incubation 37˚C. Approximately 14-15 ml of medium was added to the separated cells from surface to eliminate the effect of trypsin and they were transferred to the centrifuge tube by means of a disposable sterile pipette. The cells were centrifuged at 1500 rpm for 5 minutes to remove supernatant. The homogenized cells were subsequently seeded and an appropriate amount of new medium was added in the flasks. The flasks were incubated in 37˚C, 5% CO_2_ incubator.

### Cell proliferation assay

The cultured cells in T25 cm² flask were used for proliferation assay. The cells were removed, as indicated before. The cells were then centrifuged and suspended by the adding medium to the resultant pellet.

96-well cell culture plates were used for the experiment. Obtained from the cell suspension 90 μl of colon cells were planted in the plate. As a result of evaluations in the literature boric acid concentrations of 10, 25, 50, 75, 100 mM (28) were determined. Boric acid of the specified concentrations was dissolved in serum-free RPMI and 10 ul was added to the cells in the well. Cells that were not treated with boric acid were served as controls. For each concentration, they were planted in three wells at 24^th^ and 48^th^ hours, followed by incubation at 37˚C, 5% CO_2_ and appropriate level of moisture. 10 μl CCK-8 (ABP Biosciences, United States) was added to the wells after 24 and 48 hours and they were measured using plate reader. 

### PARP analysis

The culture cells in 75 cm² flask were used for poly (ADP-ribose) polymerase (PARP) (Sun-Red Human ELISAKits, Lot no: 201904 2, Sunredbio, China) analysis. Next the cells were detached using trypsin. They were subsequently centrifuged, supernatat was discarded and the resulting pellet were suspended in the medium.

24-well cell culture plates were used for the experiment. Colon cells were planted on the plates as 450 μl. The plate was incubated for 24 hours and at the end of 24 hours, the incubated cells from were treated with various concentrations of Boron. It was placed in a 37˚C incubator for 48 hours. After 48 hours, the cells were removed from incubator. Media were removed from the plate of cells and approximately 700 μl of PBS was added. PBS was withdrawn from the treated cells. 200 μl trypsin was added to the cells and they were put in a 37˚C incubator until cell detachment. 450 μl of medium was added to the separated cells from the surface, to eliminate effect of trypsin. The cells were transferred to the eppendorf for PARP by means of a disposable pipette.

The cells in the eppendorf were centrifuged at 2100 rpm for 20 minutes at 15˚C. After centrifugation, the supernatant was discarded. The medium was added to the eppendorf and the medium was planted on 96-well plates. Reagents, samples and standards were prepared. Prepared samples and standards were seeded on antibody-loaded 96-well plates and incubated at 37˚C for 60 minutes. Platers were bathed five times, followed by adding chromogen solution A and chromogen solution B. They were next incubated at 37˚C for 10 minutes. The stop liquor was added and measurement was performed. 

### VEGF analysis

The cultured cells in 75 cm² flask were used for VEGF (Sun-Red Human ELISA Kits, Lot no:201812, Sunredcio, China) analysis. The cells were detached using trypsin. Next, they were centrifuged, supernatat was discarded and the resulting pellet were suspended in medium.

24-well cell culture plates were used for the experiment. Colon cells were planted on the plates to 450 μl. The plate was incubated for 24 hours frollowed by treating with various concentrations of Boron. It was placed in a 37˚C incubator for 48 hours. After 48 hours, the cells were removed from the incubator. Media was removed from the plates of cells and approximately 700 μl of PBS was added to the plates. PBS was withdrawn from the treated cells. 200 μl trypsin was added to the cells and put in a 37˚C incubator util detachment. 450 μl of medium was added to the separated cells from the surface to eliminate effect of trypsin. The cells were transferred to the eppendorf ube for PARP by means of a disposable pipette.

The cells in the eppendorf were centrifuged at 2100 rpm for 20 minutes at 15˚C. After centrifugation, supernatant was discarded. The medium was next added to the eppendorf and the medium was planted on the 96-well plates. Reagents, samples and standards were prepared. Prepared samples and standards were seeded on antibody-loaded 96-well plates and incubated at 37˚C for 60 minutes. Platters were bathed five times, followed by adding chromogen solution A and chromogen solution B. They were next incubated at 37˚C for 10 minutes. The stop liquor was added and measurement was performed.

## Results

### Proliferation test

**Fig.1:**
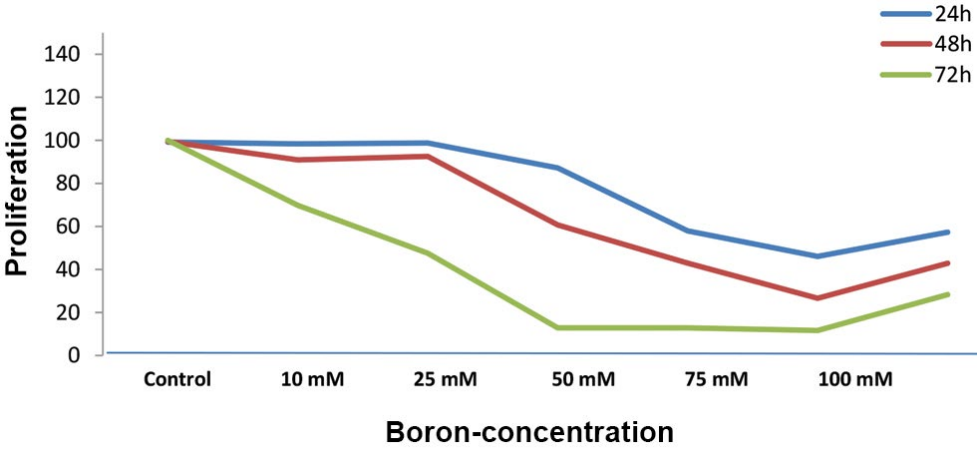
Proliferation-concentration graph. Boron concentrations given to CCL-233 cells are shown based on mM. h; hours.

Five different dose ranges were applied for CCL-233 colon cancer cell line, including 10, 25, 50, 75 and 100 mM. At the end of 24, 48 and 72 hours, the half maximal inhibitory concentration (IC_50_) was determined as 50 mM due to death of 50% of cancer cells using the CCK-8 method, while viability of the cancer cells was better at 48 hours (Fig.1). Other experiments were based on this time and dose.

### PARP and VEGF analyses 

Five different dose ranges were applied for CCL-233 colon cancer cell line, including 10, 25, 50, 75 and 100 mM. Since our experiments showed better effect after 48 hours, PARP analysis were performed at this time-priod. No significant difference was observed between boron 25 and control group. Boron 50-100 was significantly decreased, compared to the control group. PARP values were significantly increased (P<0.05) in cisplatin. VEGF analysis was carried out after 48 hours. There was no significant difference between 25 mM, 50 mM, 100 mM boric acid and control group. VEGF values were significantly decreased by cisplatin. Values are given in the table (Table 1).

**Table 1:** PARP and VEGF statistical analysis


Groups	PARP (ng/l)	VGEF (ng/l)

Control	0.3789 ± 0.06^a^	0.5161 ± 0.040ª
Boric acid (25 mmol)	0.3485 ± 0.05^a^	0.4756 ± 0.091ª
Boric acid (50 mmol)	0.1884 ± 0.004^b^	0.4568 ± 0.012ª
Boric acid (100 mmol)	0.2125 ± 0.03^b^	0.4030 ± 0.158ª
Cisplatin	0.7340 ± 0.01^c^	0.0619 ± 0.008ᵇ


Data are presented as mean ± SE. PARP; Poly (ADP-ribose) polymerase and VEGF; Vascular endothelial growth factor. Different letters in the same column are statistically significant from each other (P<0.05).

## Discussion

Boron-based compounds are studied in research on cancer treatments, due to their anticarcinogenic properties. Many studies have shown that high boron-containing media may lower the risk of some cancers such as prostate, breast, cervical and lung cancers (29). Research over the past few years has shown that boron compounds as anticancer agents are used extensively, particularly in non-operative cancers and high malignant cancers (30). 

The purpose of this study is to search influence of different concentrations of boric acid on colon cancer cells (CCL-233) *in vitro* conditions, which are common in the world and our country.

Wade and Eckhert (30) investigated inhibition of boric acid on human prostate cancer cell proliferation. They used human prostate cancer HTB-81, CRL-1740 and CRL-1435 cell lines in their studies. For eight days, the medium containing 0-1000 μM boric acid was added to these cells and the cells were monitored. subsequentely, proliferation, apoptosis, cell cycle and mitochondrial activation were evaluated in the cells. It was observed that administration of boric acid clearly reduced dose-dependently cell proliferation in the utilized DU145, LNCaP and PC3 cancer cell lines. In another study, Scorei et al. (31) investigated effects of boric acid and calcium fructobate on HTB-26 breast cancer cell line. Efficacy of different doses of boric acid and fructoborate (0.45-22.5 mM) on cell viability were investigated using MMT assay. Calcium fructoborate and boric acid showed concentration-connected cytotoxicity in HTB-26 cells. In a study in CCL-228-SW480 colon cancer cells, impact of boric acid on cancer cells were investigated. Effect of adminsterated boric acid (10-100 mM) at different doses were investigated on cell viability after 24, 48 and 72 hours using BrdU method. As a result of the evaluations, it was observed that there was meaningful decrease in the count of cells given boron, against the control group at the applied hours (28). 

As a result of our analysis, when the molecular properties of CCL-233 cell lines for the proliferation assay were evaluated in the light of the literature findings, dose ranges of these cells were determined as 10 mM, 25 mM, 50 mM, 75 mM and 100 mM. Doses acting on the same substance are different in various cancer cell lines. The reason for this was interpreted as there might be differences in the molecular biology of each cancer cell line. A direct proportional decrease in cell proliferation was observed in the CCL-233 colon cancer cell line at 24, 48 and 72 hours. However, after a very high antiproliferative effect in the 72^th ^hour data, other studies were conducted over a 48 hour incubation period. When the proliferation results were evaluated, IC50 dose was 50 mM and this dose was used in the experiments. Findings showed significant decrease in proliferation of the treated cells with boric acid. Our findings are consistent with the findings of many previous studies.

PARP is a nucleus enzyme, highly presented in eukaryotes and activated in response to DNA damage. PARP overactivation leads to more NAD⁺ and ATP consumption, which in turn disrupts cell function or causes necrosis. As a result of our analysis, PARP levels were investigated in order to determine potential of apoptotic effect of boric acid. In these findings, PARP values ​​were significantly higher in cisplatin. This was interpreted as causing cell necrosis, due to the overactivation of PARP in cisplatin, as a consequence of increased NAD and ATP consumption. In another study, DU-145 (HTB-81) human prostate cancer cells were analyzed to determine apoptotic effect of boric acid, when medium containing 0-1000 mM boric acid was added for eight days. Western blot analysis of boric acid exposed cells showed no caspase-3. Using protein immunoblot, cell loop assay of HTB-81 cells indicated that boric acid attached proliferative inhibition did not lead to phase change in a marked cell cycle. Since there is often a deviation in the cell cycle during apoptosis, this inhibition of cell proliferation was demonstrated to occur in the absence of apoptosis. These results are supported by the fact that caspase-3 cannot be detected in Western blot technique and no finding is found in DNA fragmentation analysis.

These conclusions backing the commentary that boric acid reduces growth by inmhibiting proliferation instead of inducing apoptosis (31). Caspase-3, apoptosis induction factor (AIF) and TUNEL assays were used in another study to determine apoptotic effect of boric acid. Cancerous cells, except for 75 mM boric acid-containing cells, showed that a small number of cells underwent apoptosis in all methods (caspase-3, AIF, TUNEL) compared to the control group at the time of administration. However, CCL-228-SW-480 colon cancer cells were applied with 75 mM boric acid; it was found that many cells underwent apoptosis at all analyzed times compared to the control group. This showed that 75 mM boric acid caused apoptotic DNA fragmentation and apoptosis in CCL-228-SW-480 cells (28). Gambi et al. (32) investigated the PARP activity of cisplatin. HT29 cells were exposed to 10 mM cisplatin for four hours. Cisplatin showed a time-dependent activation of PARP, leading to two-fold increase in activation of this enzyme, 48 hours after the procedure. They evaluated PJ34 inhibitory activity in the same assay. Findings showed that 1 mM PJ34 inhibitor was able to eliminate almost (<12%) enzymatic activity by adding to the reaction mixture.

As a result of our analysis, PARP (an enzyme activated by DNA break) levels were significantly higher in cisplatin-treated cells than the control group. Cisplatin caused an excessive increase in PARP activity. PARP activation modulates important inflammatory pathways. PARP overactivation depletes its substrate (NAD), slows down the rate of glycolysis, electron transport and ATP formation and ultimately leads to cell death. consequently, PARP leads to necrosis by increasing consumption of NAD and ATP due to the overactivation of PARP in cisplatin. In our study, it was concluded that excessive increase in PARP levels in cisplatin led to cell necrosis. The results of this study suggested that overactivation of PARP in cisplatin led to cisplatin nephrotoxicity. In contrast, PARP levels were low at two boron levels (50-100 mM). Low levels of PARP in boron led us to think that the PARP enzyme was blocked in the cells treated with boric acid. In conclusion, our experience suggested that boron disrupted structure of the PARP enzyme or might function as a PARP inhibitor. 

Cancer cells grow and multiply uncontrolled after a certain time. These cells need new vascular formation to grow further. Cancer cells secrete a number of angiogenic factors (VEGF, EGF and interleukin-18). Many of these factors act on different small vessels and bind to receptors in the endothelial cell, resulting in new vessels. Based on this information, VEGF levels increase in cancer cells and decrease in cytotoxic drugs. Studies investigated effect of boron on treatment process of boron derivatives in human testicular germ cell tumors. It was found to be able to produce an anti-invasive effect by suppressing VEGF expression at high doses (33).

As a result of our analysis, a method not performed in other studies was applied. VEGF levels were measured in the colon cancer cell line treated with boric acid separated according to their concentrations. Three concentrations of boron and cisplatin were used according to different concentrations. These boron levels and cisplatin were evaluated together with the control group. As a result of the statistical analyses, it was found that there was a significant difference between the levels of boron and cisplatin, but no significant difference was determined compared to the control groups. In our study, cisplatin caused a decrease in VEGF expression. It showed that boron did not cause any change in VEGF expression level compared to the control group. Another result of this study showed that boric acid did not affect VEGF expression level.

## Conclusion

As a result, we can say that various boric acid concentrations have antiproliferative effect on CCL-233 colon cancer cell line. We propose that increased levels of PARP in cisplatin lead to cell necrotic death. Boric acid may disrupts structure of PARP enzyme or it may be like a PARP inhibitor. The cells in the cisplatin were less active. Boric acid VEGF showed no effect on expression level. On the other hand, there are many unknown subjects about the formation mechanism of colon cancer. It is belived that the molecular mechanisms leading to this type of cancer as well as the effect of boric acid on colon cancer should be clarified in more detailed ways for the early diagnosis and treatment of colon cancer.
